# Deep learning-based identification of causative genes in lissencephaly using 3D-MRI volumetric datasets

**DOI:** 10.1016/j.ynirp.2026.100375

**Published:** 2026-06-23

**Authors:** Naoki Takahashi, Yoshihiro Sato, Mitsuhiro Kato, Atsuko Yamaguchi

**Affiliations:** a Department of Design and Data Science, Tokyo City University, Kanagawa, Japan; b Department of Pediatrics, SHOWA Medical University, Tokyo, Japan

**Keywords:** Neuronal migration disorders, Lissencephaly, MRI, Medical image processing, Deep learning, 3D-CNN

## Abstract

**Background:**

This paper reports a genetic identification task using 3D convolutional neural network (3D-CNN) models applied to a proprietary 3D magnetic resonance imaging (MRI) dataset of patients with lissencephaly. Lissencephaly is a neuronal migration disorder caused by genetic mutations or deletions in which specific causative genes result in distinct morphological alterations in brain structure.

**Objective:**

The objective of this study was to identify causative genes through image classification by analysing three-dimensional structural features of brain MRI using deep learning.

**Methods:**

In our experiments, we extended representative CNN architectures to handle three-dimensional inputs and performed three-class classification targeting the primary causative genes, *LIS1* and *DCX*, along with a category for other genetic variations.

**Results:**

Our results demonstrated that the 3D-ResNet18 model achieved a mean classification accuracy of over 78%. Furthermore, to enhance the precision for primary genes, we introduced a decision-making process based on prediction probability thresholds.

**Conclusions:**

This approach yielded an average precision improvement of 4.67% for *DCX* and 4.84% for *LIS1* across all the evaluated models.

## Highlights

• 3D-CNN models trained on brain MRI identified causative genes in lissencephaly.
• 3D-ResNet18 achieved an accuracy of 78.4% in a three-class classification task.
• A prediction probability threshold of 0.9 improved mean precision.
• Grad-CAM visualizations revealed holistic brain responses during feature extraction.
• 15/78 trained models correctly identified both T1-weighted and T2-weighted images.

## 1 Introduction

Lissencephaly is a neuronal migration disorder (NMD) caused by mutations or deletions in specific genes. NMDs primarily affect brain formation during the foetal period and encompass a range of conditions, including lissencephaly, polymicrogyria, and heterotopia (Barkovich et al., 2012). Lissencephaly, in particular, is a major type of NMD. As a primary manifestation within this spectrum, it has been extensively studied in the fields of pathology and medical genetics (Kato and Dobyns, 2003; Forman et al., 2005). In brains affected by lissencephaly, the cerebral gyri and sulci are reduced, resulting in a characteristic ‘smooth’ surface. These morphological features can be visually identified using medical imaging modalities, such as brain magnetic resonance imaging (MRI). Genetic research using brain MRI has identified several causative genes, including *LIS1*, *DCX*, and *TUBA1A* (Reiner et al., 1993; des Portes et al., 1998; Keays et al., 2007). Furthermore, specific morphological features appearing on the brain surface differ depending on the causative genes involved (Pilz et al., 1998). However, because of its rarity and impact on foetal development, which can lead to intrauterine growth restriction or foetal death, acquiring brain MRI data is difficult. Consequently, there are inherent limitations to performing large-scale analyses of these morphological characteristics.

In recent years, deep learning has gained prominence as a processing technology that enables more complex and sophisticated analyses than traditional machine-learning methods. It has garnered significant attention, particularly because of breakthrough improvements in image-processing accuracy (Krizhevsky et al., 2017). In the domain of computer vision, convolutional neural networks (CNNs) are among the most frequently employed deep-learning architectures, and numerous studies have applied CNN-based models to medical image processing (Litjens et al., 2017; Mienye et al., 2025). While most conventional image-processing models are designed for feature extraction from two-dimensional (2D) images, brain MRI is three-dimensional (3D) and requires holistic capture of spatial and volumetric features. For such 3D images, models incorporating 3D-CNN processes, which include 3D convolutional operations, can extract features that capture complex three-dimensional structural information associated with disease-specific morphologies. However, research on 3D image-processing models trained on 3D MRI for lissencephaly remains limited.

Therefore, in this study, we collaborated with hospitals across Japan to aggregate lissencephaly data. As a foundational step toward large-scale data analysis, we aimed to construct a classification task for identifying causative genes using a brain MRI dataset of patients with lissencephaly. We hypothesised that 3D-CNN models could capture holistic morphological features directly from volumetric MRI data and accurately differentiate between primary causative genes (*LIS1* and *DCX*) and other genetic variations.

## 2 Methods

In this study, we performed causative gene identification focusing on mutations in *LIS1* and *DCX*—the primary factors associated with classical lissencephaly—using a brain MRI dataset. Our objective was to computationally capture the morphological features associated with different genetic factors and clarify the data-driven results of genetic identification.

### 2.1 Brain MRI dataset

The brain MRI dataset used in this analysis was compiled from clinical cases of lissencephaly across Japan, centred on Showa Medical University and its affiliated medical institutions. The collection of this dataset was approved by the Institutional Review Board (IRB) for Research Involving Human Subjects at Showa Medical University, and informed consent was obtained from all participants or their legal guardians.

The collected brain MRI scans consisted of three-dimensional image data of the patients’ heads. Each case included metadata regarding the causative genes and MRI acquisition parameters. The dataset contained approximately 180 cases with confirmed causative genes and approximately 260 cases in total, including unconfirmed instances. Among the gene-confirmed cases, the majority involved mutations in *LIS1* or *DCX*, which are the primary causes of classical lissencephaly; 60 cases specifically involved mutations in these two genes. The dataset recorded more than 20 different types of genetic abnormalities. A detailed breakdown of these specific genetic variations and their corresponding case counts is provided in Supplementary Table S1. The MRIs were primarily acquired using two modalities: T1-weighted (T1w) and T2-weighted (T2w) imaging; other sequences, such as Fluid-Attenuated Inversion Recovery (FLAIR), were not included. Thus, the dataset maintained quantitative consistency, which allowed determination of the correspondence between disease morphology and genetic information through image classification.

### 2.2 Construction of the causative gene identification model

We implemented a gene identification task using an image-processing model centred on 3D-CNN processing. Similar to standard 2D-CNNs, the 3D-CNN mechanism performs convolution operations on 3D spatial dimensions using 3D kernels of a specified size. We designed our 3D image classification model by incorporating this mechanism into the architectures of 2D image classification models originally utilised for ImageNet (Deng et al., 2009). When designing the architecture, all components, including convolutional layers, pooling layers, and other standard 2D processing units, were converted to their 3D counterparts. This conversion allows the construction of a classification model adapted for 3D images while maintaining the fundamental structure of general image classification models used for natural images.

To perform gene identification using these 3D-CNN models, we trained them on a 3D MRI dataset of patients with lissencephaly. The model was configured for a three-class classification task: *LIS1* gene mutations, *DCX* gene mutations, and ‘Other’ genetic abnormalities. This approach enabled the identification of the two primary genetic causes of classical lissencephaly based on the unique morphological features captured on each patient's brain MRI.

### 2.3 Precision optimisation via thresholding

To ensure reliable identification and minimise false positives, we implemented a classification strategy applying prediction probability thresholds. Rather than determining a single fixed threshold based on prior clinical references or empirical experience, we conducted a purely data-driven parametric evaluation by varying the threshold from 0.1 to 0.9 in discrete increments. This approach was designed to characterise the trade-off between prediction volume and precision, illustrating how filtering low-confidence predictions can improve identification accuracy. A prediction of *LIS1* or *DCX* was assigned only if the probability exceeded the threshold and was the highest among all classes. If the threshold was not met, the case was categorised as ‘Other’. This comprehensive evaluation ensures that the reported trends reflect the model's inherent confidence–accuracy relationship without data leakage.

### 2.4 Training and evaluation

To train the causative gene identification models, 181 gene-confirmed cases were divided into training and validation sets at a ratio of 5:1. The dataset was balanced across the three classes, with 60 cases each for *LIS1* and *DCX*, and 61 cases for ‘Other’ genetic abnormalities. Each class contained an approximately equal distribution of T1w and T2w images. Because this study utilised a multicentre dataset collected from various paediatric hospitals across Japan, the raw MRI acquisition parameters—including scanner manufacturers (e.g., GE, Philips, Siemens), magnetic field strengths (1.5 T and 3.0 T), echo time (TE), repetition time (TR), field of view (FOV), and slice thickness—naturally varied according to each institution's distinct clinical protocols. To ensure the model's robustness and generalisability across these heterogeneous real-world conditions without bias toward a specific scanner, standardising the acquisition parameters at the time of scanning was not feasible. As a preprocessing step, all 3D MRI data were resized to a uniform resolution of 64 × 128 × 128 voxels using trilinear interpolation (order = 1 in SciPy ndimage.zoom) before being input into the models. To preserve the original spatial relationships and morphological scaling of the congenital malformations, data augmentation techniques (e.g., rotations or scaling) were intentionally omitted. Notably, standard neuroimaging preprocessing steps—such as N4 bias field correction, intensity normalisation, and skull-stripping—were not applied in this foundational study. The models were trained directly on the resized raw volumes to evaluate the baseline capability of the 3D-CNNs to extract features from unaltered clinical scans. All computational experiments and model training were implemented using TensorFlow (version 2.12.0) in a Python 3.10.18 environment accelerated by NVIDIA RTX PRO 6000 Blackwell Max-Q Workstation Edition GPUs.

We evaluated 13 model architectures, including various configurations of VGGNet (Simonyan and Zisserman, 2015) and ResNet (He et al., 2016) adapted for 3D classification to investigate the effects of depth and structure. Training was conducted using minibatch learning with a batch size of eight, and categorical cross-entropy was employed as the loss function. We used the Adam optimiser (Kingma and Ba, 2015) with a learning rate policy incorporating a warm-up period and a cosine decay scheduler. Over 200 epochs, the learning rate started at 1 × 10^−4^, rose to 1 × 10^−3^ by epoch 5, and then gradually decayed to zero following the cosine schedule. This strategy was implemented to stabilise the learning process and mitigate overfitting.

Given the inherent sample-size limitations associated with rare diseases, allocating a separate hold-out test set would have severely diminished the training data. Therefore, model performance was evaluated using 6-fold stratified cross-validation to maximise data utilisation while providing an unbiased performance estimate. In addition to overall accuracy across folds, we used macro-averaged precision, recall, and F1-score as evaluation metrics. The results were obtained using the model parameters that achieved the highest validation accuracy during training.

For the gene identification task involving threshold settings, we evaluated the results by averaging the precision of *LIS1* and *DCX* predictions across all folds. Furthermore, the total number of predictions for each gene was calculated as the average per model across folds. To investigate the effect of thresholding on precision, we varied the threshold from 0.1 to 0.9 in increments of 0.1 and recorded the resulting precision to characterise performance trends across different confidence levels.

To identify the specific features within the MRI volumes used by the trained models for classification, we generated heat maps using gradient-weighted class activation mapping (Grad-CAM). Grad-CAM is used to visualise the regions that contribute most significantly to the prediction results of CNN-based models (Selvaraju et al., 2017). In this study, we generated heat maps for correctly classified images in each genetic class using ResNet18, which demonstrated the highest accuracy.

Although Grad-CAM typically uses gradient information from feature maps in the final convolutional layer, Selvaraju et al. (2017) noted that it can be effectively applied to any layer. In our models, the final output layer had low spatial resolution, which led to substantial loss of structural information. Therefore, we extracted gradients from an earlier CNN layer with an output size of 8 × 16 × 16 to calculate contributions to the prediction while preserving better spatial localisation.

## 3 Results

Table 1 summarises the macro-averaged accuracy, precision, recall, and F1-score for each evaluated model. Values are presented as the mean ± standard deviation across the cross-validation folds. Among all architectures, 3D-ResNet18 achieved the highest performance, with a mean accuracy of 78.4%.

**Table 1:** Performance metrics for each model.

Model	Accuracy	Precision	Recall	F1-score
VGG16 (Simonyan and Zisserman, 2015)	0.729 (±0.093)	0.742 (±0.092)	0.73 (±0.094)	0.723 (±0.095)
VGG19	0.674 (±0.107)	0.706 (±0.108)	0.675 (±0.107)	0.655 (±0.123)
ResNet18 (He et al., 2016)	0.784 (±0.086)	0.802 (±0.077)	0.783 (±0.086)	0.781 (±0.088)
ResNet34	0.778 (±0.092)	0.801 (±0.08)	0.778 (±0.092)	0.778 (±0.09)
ResNet50	0.712 (±0.061)	0.742 (±0.074)	0.712 (±0.06)	0.714 (±0.059)
ResNet101	0.745 (±0.07)	0.78 (±0.073)	0.744 (±0.071)	0.743 (±0.074)
ResNet152	0.746 (±0.062)	0.773 (±0.044)	0.746 (±0.062)	0.746 (±0.06)
WideResNet-16-4 (Zagoruyko and Komodakis, 2016)	0.756 (±0.097)	0.771 (±0.092)	0.757 (±0.097)	0.754 (±0.097)
DenseNet121 (Huang et al., 2017)	0.712 (±0.083)	0.742 (±0.16)	0.711 (±0.083)	0.696 (±0.12)
ResNeXt50 (Xie et al., 2017)	0.74 (±0.07)	0.76 (±0.072)	0.74 (±0.07)	0.731 (±0.075)
MobileNetV1 (α**=**1.00) (Howard et al., 2017)	0.718 (±0.073)	0.748 (±0.074)	0.717 (±0.073)	0.713 (±0.078)
MobileNetV2 (α**=**1.00) (Sandler et al., 2018)	0.718 (±0.049)	0.762 (±0.046)	0.717 (±0.049)	0.699 (±0.065)
Xception (Chollet, 2017)	0.712 (±0.062)	0.744 (±0.069)	0.714 (±0.062)	0.709 (±0.064)

Average	0.733 (±0.077)	0.759 (±0.082)	0.733 (±0.077)	0.726 (±0.084)

To further assess performance on the imbalanced dataset, class-specific metrics were calculated for each gene category (Table 2). Although performance varied across architectures, 3D-ResNet18 and ResNet34 consistently achieved high Area Under the Precision–Recall Curve (AUPRC) values across all classes. Specifically, 3D-ResNet18 achieved AUPRC scores of 0.753 for *DCX*, 0.816 for *LIS1*, and 0.837 for Others.

**Table 2:** Per-class F1-score and AUPRC for each model.

Model	F1-score (DCX)	F1-score (LIS1)	F1-score (Others)	AUPRC (DCX)	AUPRC (LIS1)	AUPRC (Others)
VGG16	0.729	0.753	0.688	0.730	0.717	0.752
VGG19	0.562	0.717	0.687	0.654	0.624	0.679
ResNet18	0.740	0.790	0.813	0.753	0.816	0.837
ResNet34	0.736	0.785	0.813	0.793	0.828	0.895
ResNet50	0.721	0.737	0.684	0.729	0.757	0.745
ResNet101	0.732	0.747	0.761	0.756	0.781	0.768
ResNet152	0.687	0.732	0.728	0.708	0.718	0.708
WideResNet-16-4	0.763	0.748	0.753	0.803	0.799	0.837
ResNeXt50	0.753	0.685	0.757	0.771	0.754	0.743
DenseNet121	0.646	0.725	0.718	0.795	0.785	0.808
MobileNetV1	0.648	0.706	0.745	0.687	0.755	0.702
MobileNetV2	0.695	0.724	0.709	0.747	0.734	0.751
Xception	0.641	0.767	0.731	0.676	0.753	0.730

**Fig. 1:**
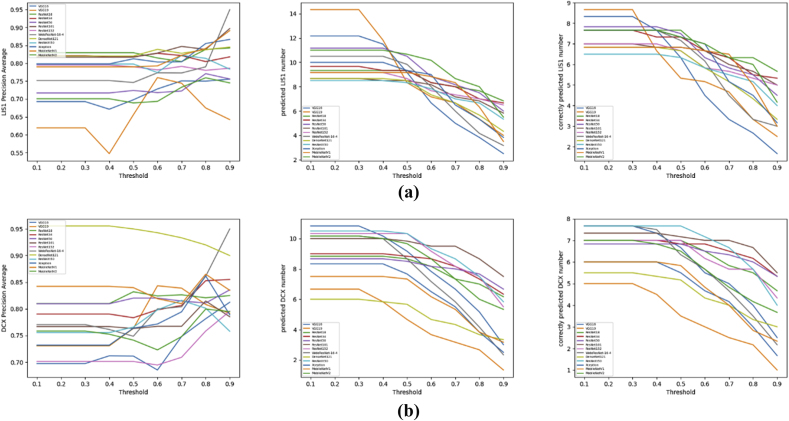
**Impact of prediction probability thresholds on model performance.** The graphs show trends in classification precision (left), total number of predictions (middle), and number of correctly predicted samples (true positives; right) as the probability threshold increases from 0.1 to 0.9 across various 3D-CNN models. (a) Results for the *LIS1* class. (b) Results for the *DCX* gene class. Higher thresholds generally improve precision but reduce the numbers of predictions and correct identifications, highlighting the trade-off between prediction confidence and coverage.

Fig. 1 shows the effects of probability thresholding. Fig. 1(a) and (b) present the results for the *LIS1* and *DCX* classes, respectively. Increasing the threshold to 0.9 improved mean precision (left panels) by 4.84% for *LIS1* and 4.67% for *DCX* across all models. Concurrently, the middle and right panels show the model-wise trends of the total number of predictions and the number of correct predictions, respectively. Both measures decreased as the confidence threshold increased.

**Fig. 2:**
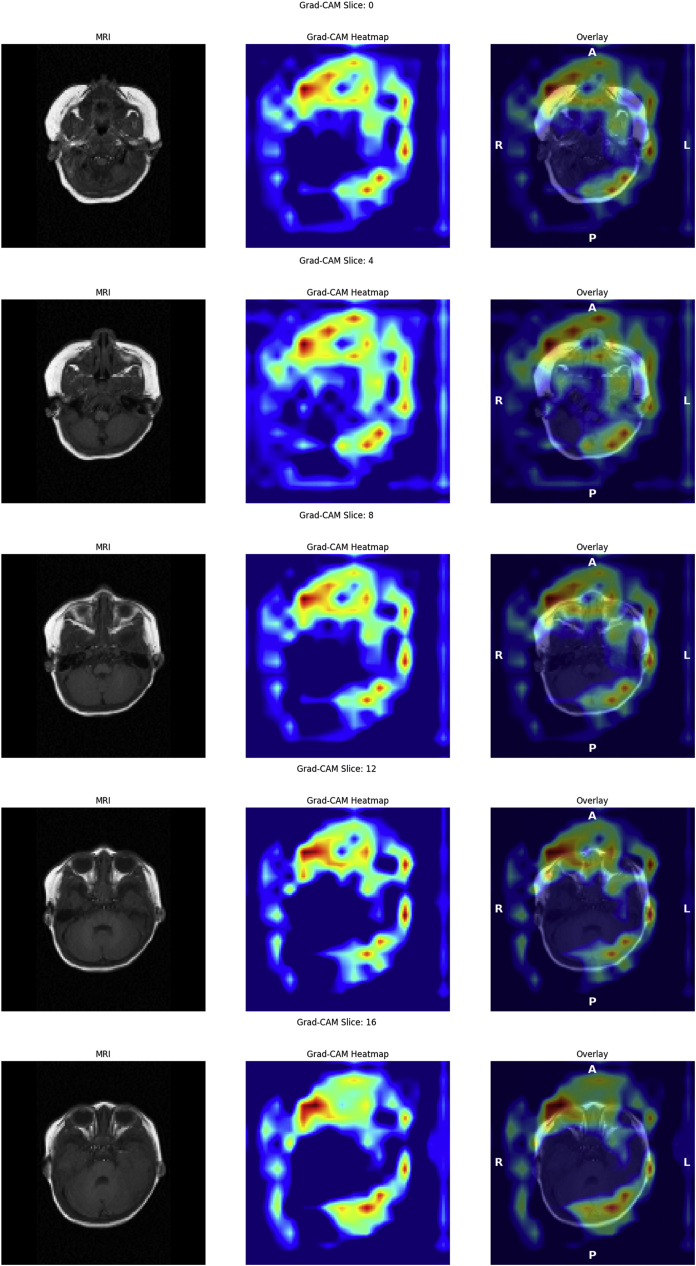

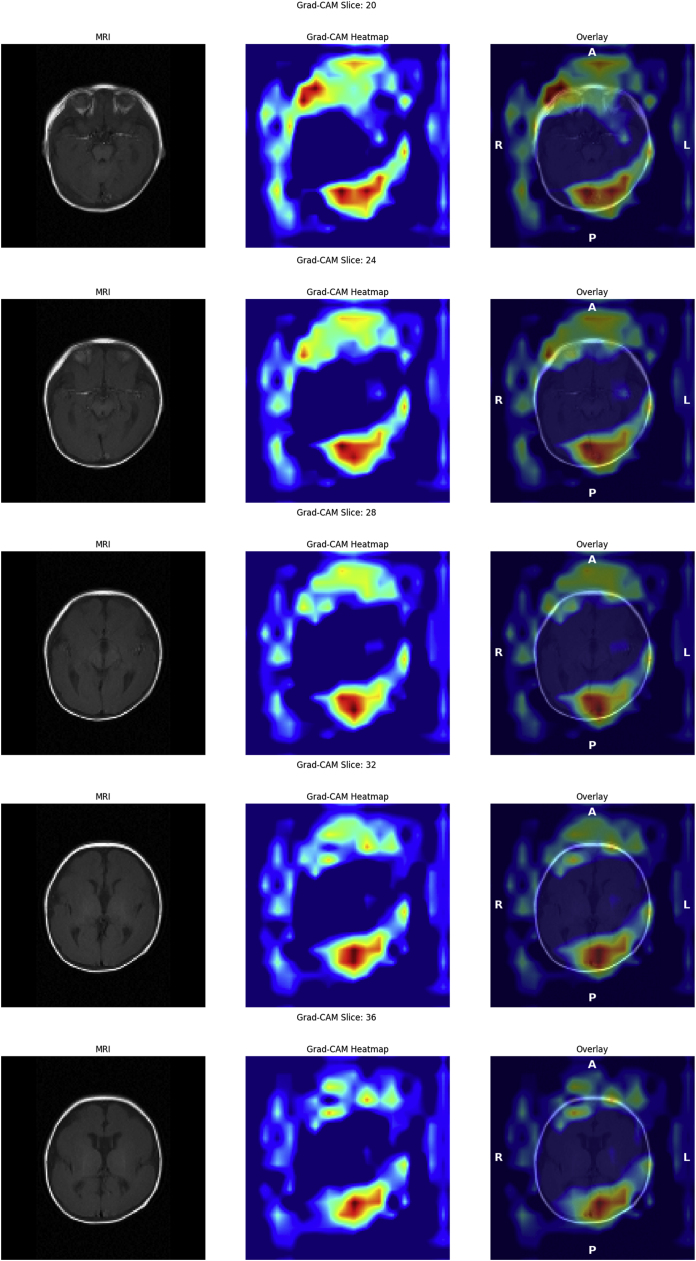

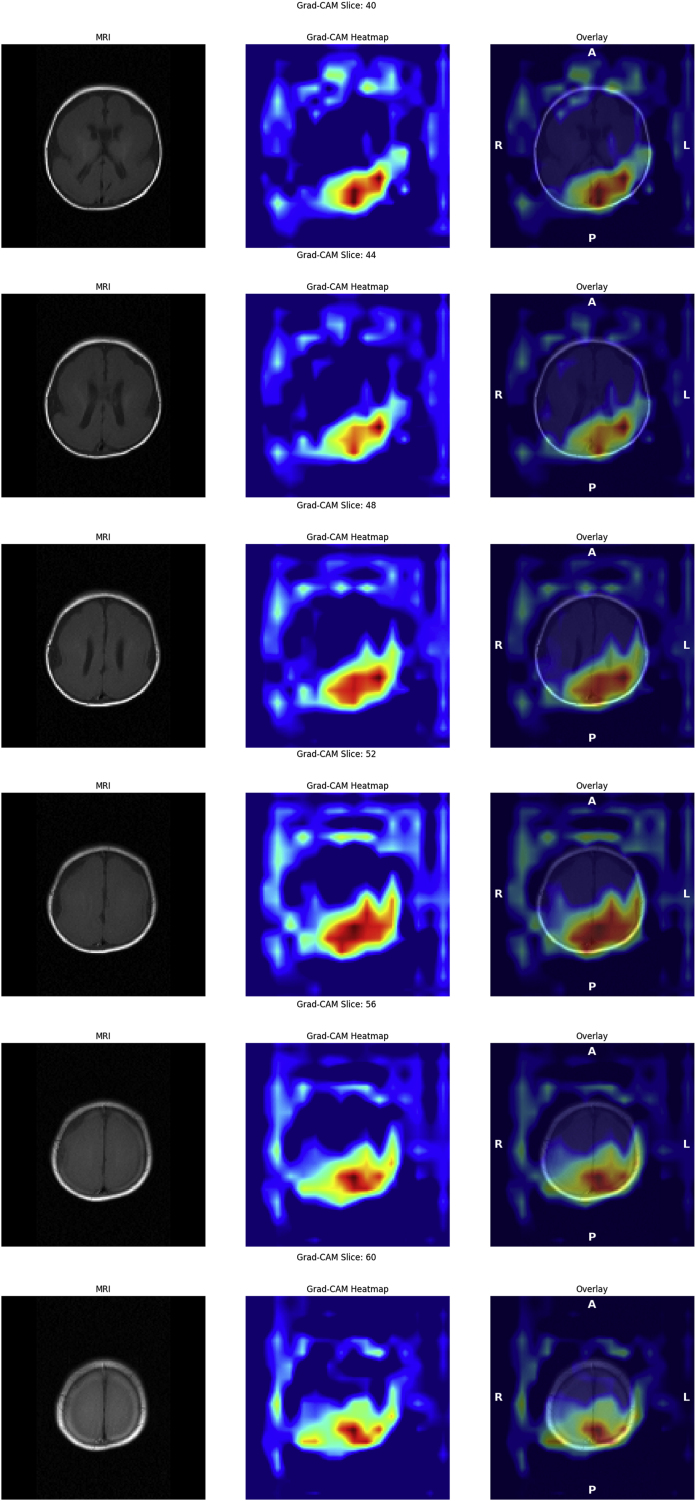
**Grad-CAM visualisation of feature-extraction areas for the *LIS1* class**. Heatmaps generated using the trained 3D-ResNet18 model are overlaid on the original MRI data to indicate regions contributing to the classification. Warmer colours (red/yellow) indicate higher contribution scores. The visualisation displays a montage of 16 representative axial slices spanning the entire brain volume from superior to inferior regions, illustrating the distribution of the model activation.

**Fig. 3:**
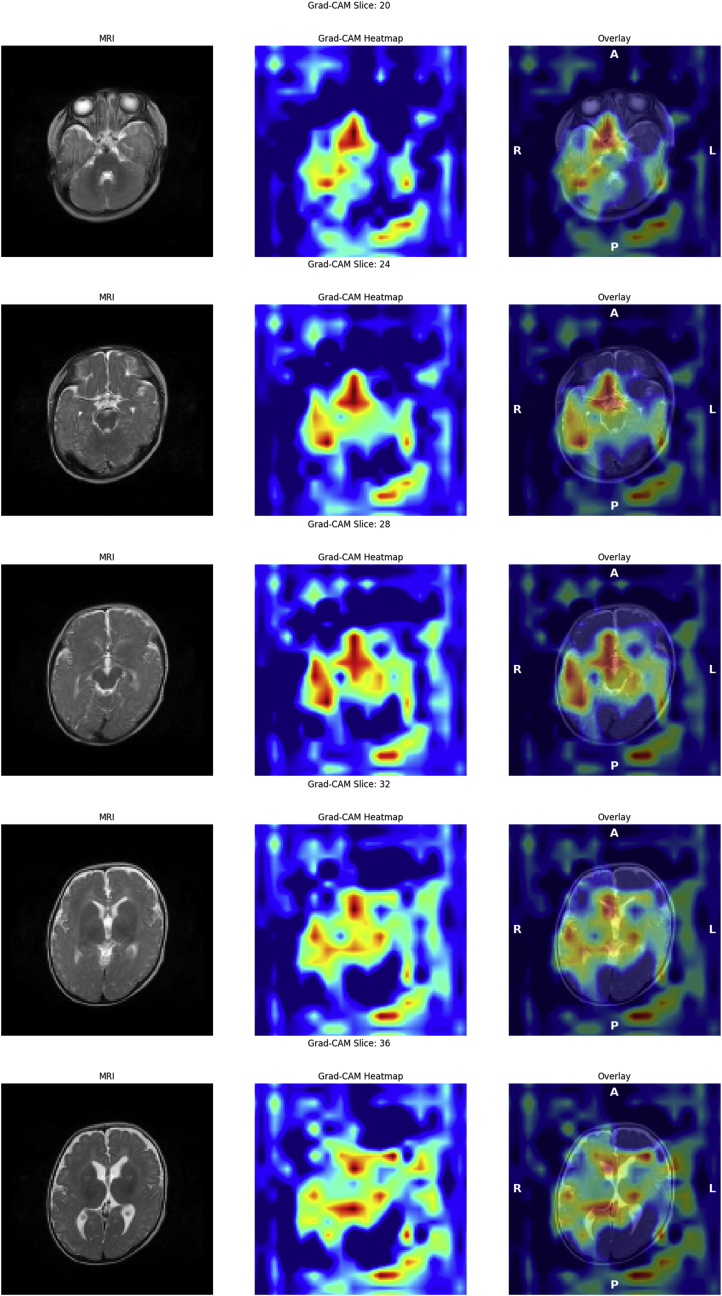

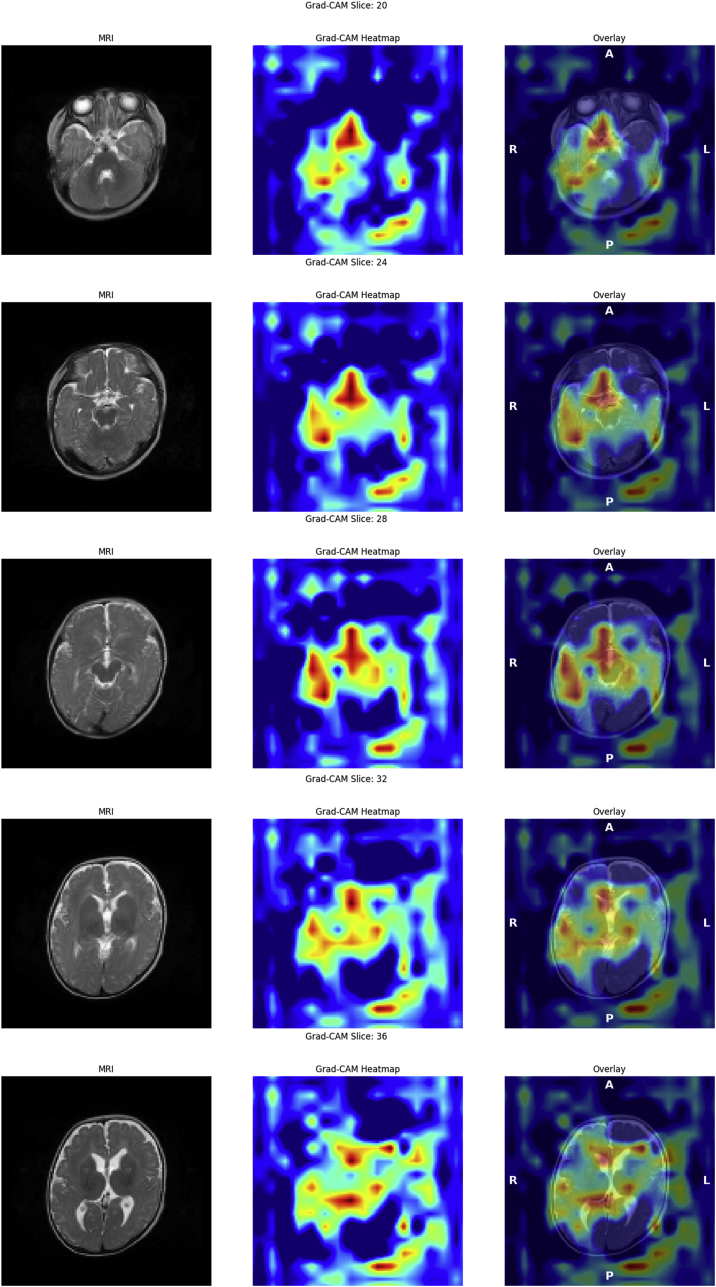

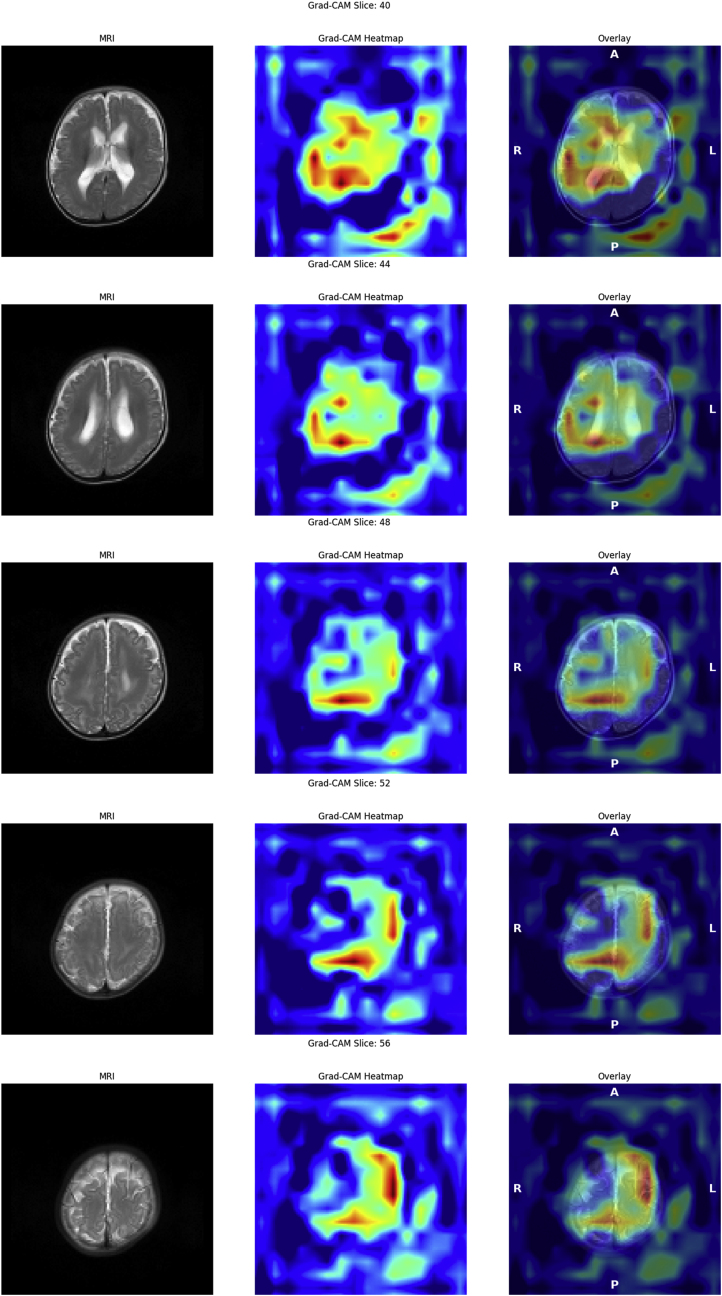
**Grad-CAM visualisation of feature-extraction areas for the *DCX* class.** Heatmaps generated using the trained 3D-ResNet18 model are overlaid on the original MRI data to indicate regions contributing to the classification. Warmer colours (red/yellow) indicate higher contribution scores. The visualisation presents a montage of 16 representative axial slices spanning the brain volume from superior to inferior regions, illustrating the distribution of model activation.

Fig. 2, Fig. 3 present Grad-CAM heat maps for the *LIS1* and *DCX* classes, respectively, highlighting the regions contributing to model predictions. Each figure includes 16 representative axial slices spanning the entire brain volume from the superior to inferior regions. Visual inspection revealed that high-contribution regions were distributed throughout the brain rather than concentrated in specific localised areas, indicating that the models utilised broadly distributed features for classification.

### 3.1 External validation using an open-data sample

To evaluate model generalisability, we applied the trained models to a publicly available brain MRI scan from a 5-year-old girl with lissencephaly type 1 and subcortical band heterotopia (SBH), and a genetically confirmed DCX deletion (Chieng, 2024). Both T1w and T2w 3D MRI volumes were subjected to the same preprocessing pipeline used in the primary experiments.

Table 3, Table 4 summarise the genetic identification results across 78 trained models (13 architectures × 6 folds) for the T1w and T2w MRI scans, respectively. For the T1w images, 30 models identified the case as *DCX*, 12 as *LIS1*, and 36 as ‘Other’. For the T2w images, 23 models identified it as *DCX*, 21 as *LIS1*, and 34 as ‘Other’. Overall, 15 models (19.2%) correctly identified *DCX* mutations using both imaging modalities. Among the evaluated architectures, VGG16 demonstrated the highest consistency, correctly identifying the mutation in five of six folds for both modalities.

**Table 3:** Open-data prediction results (T1-weighted MRI).

Model	Fold1	Fold2	Fold3	Fold4	Fold5	Fold6
VGG16	*LIS1*	*DCX*	*DCX*	*DCX*	*DCX*	*DCX*
VGG19	*LIS1*	*DCX*	*LIS1*	*DCX*	*LIS1*	*DCX*
ResNet18	Others	Others	Others	Others	*DCX*	Others
ResNet34	Others	Others	Others	*DCX*	*LIS1*	*LIS1*
ResNet50	Others	Others	Others	*DCX*	*DCX*	Others
ResNet101	*DCX*	*DCX*	*DCX*	*DCX*	Others	*DCX*
ResNet152	Others	Others	*DCX*	*DCX*	*DCX*	Others
WideResNet-16-4	*DCX*	*DCX*	Others	*LIS1*	*LIS1*	*DCX*
DenseNet121	Others	*DCX*	*LIS1*	Others	Others	*DCX*
ResNeXt50	Others	Others	Others	Others	*DCX*	Others
MobileNetV1 (α**=**1.00)	Others	Others	Others	Others	*DCX*	*LIS1*
MobileNetV2 (α**=**1.00)	Others	*LIS1*	Others	Others	*DCX*	*LIS1*
Xception	Others	Others	*DCX*	Others	*DCX*	Others

**Table 4:** Open-data prediction results (T2-weighted MRI).

Model	Fold1	Fold2	Fold3	Fold4	Fold5	Fold6
VGG16	*LIS1*	*DCX*	*DCX*	*DCX*	*DCX*	*DCX*
VGG19	*LIS1*	*DCX*	*LIS1*	*LIS1*	*LIS1*	*LIS1*
ResNet18	Others	Others	*DCX*	Others	*DCX*	*DCX*
ResNet34	Others	Others	Others	*DCX*	*LIS1*	*DCX*
ResNet50	Others	*DCX*	*DCX*	*DCX*	*DCX*	*DCX*
ResNet101	*DCX*	Others	Others	Others	*DCX*	*LIS1*
ResNet152	*LIS1*	Others	*DCX*	*LIS1*	*DCX*	Others
WideResNet-16-4	*LIS1*	Others	Others	*LIS1*	*LIS1*	*LIS1*
DenseNet121	Others	Others	*LIS1*	Others	Others	Others
ResNeXt50	Others	Others	*DCX*	Others	Others	Others
MobileNetV1 (α**=**1.00)	Others	*LIS1*	*LIS1*	Others	Others	Others
MobileNetV2 (α**=**1.00)	*LIS1*	*LIS1*	Others	Others	*LIS1*	*LIS1*
Xception	Others	Others	*DCX*	Others	*DCX*	Others

## 4 Discussion

In the performance comparison across the models shown in Table 1, the classification model based on ResNet18, a relatively small architecture, achieved the highest accuracy, outperforming deeper models such as ResNet50 and advanced architectures such as WideResNet and DenseNet. This finding contrasts with the general trend that increasing network depth improves classification performance. However, the limited dataset size may partly explain this result. Each class contained approximately 60 cases, substantially fewer than large-scale datasets, such as ImageNet, which comprises over 10 million images. Furthermore, to prevent the loss of spatial relationships between disease-specific features, data augmentation techniques (e.g., rotations or reflections) were intentionally omitted during training. Unlike natural images, which contain variable object orientations and viewing angles, brain MRIs are acquired in standardised planes (e.g., axial cross-sections) and provide spatially consistent structural information. Because the input orientation is highly standardised, CNN models do not necessarily need to learn the rotational or viewpoint invariance typically required for natural images. Therefore, extremely deep architectures may not be necessary for this task. This inherent structural consistency may explain why geometric augmentation was unnecessary yet yielded highly discriminative features on our small-scale dataset. Furthermore, it provides a possible explanation for why the relatively shallow 3D-ResNet18 model achieved high accuracy while avoiding the overfitting observed in deeper models.

Furthermore, while advanced architectures such as Transformers and Mamba-based models have demonstrated strong performance in large-scale computer vision tasks, their application to 3D medical imaging for rare diseases remains challenging. Unlike CNNs, which possess inherent inductive biases that facilitate learning from limited datasets, attention-based models often require significantly larger cohorts or extensive pre-training to outperform established CNN architectures. Given the rarity of lissencephaly and the need for clinical interpretability through spatial feature mapping (e.g., Grad-CAM), we focused on benchmarking 3D-CNNs as a foundational approach. Future studies will evaluate Transformer- and Mamba-based models pre-trained on large-scale neuroimaging datasets (e.g., ADNI or UK Biobank) to determine their utility for causative gene identification in lissencephaly.

The threshold-based classification (Fig. 1, left panels) demonstrated that increasing the prediction probability threshold to 0.9 improved precision across all models by an average of 4.84% for *LIS1* and 4.67% for *DCX*. Notably, WideResNet showed increases of 19.83% for *LIS1* and 17.95% for *DCX*, reaching a peak precision of 0.95 for both genes. These findings indicate that high thresholds can reduce low-confidence misclassifications and improve the reliability of genetic identification.

However, the numbers of predictions and correct identifications (Fig. 1, middle and right panels) decreased substantially as the threshold increased. For WideResNet, at a threshold of 0.9, the mean number of correct predictions dropped by 5.5 points to 2.16 for *LIS1* and by 4.66 points to 3.00 for *DCX*. Because thresholding creates a larger disparity in the number of correct identifications compared with standard classification, the limited range in which genetic identification can be confidently performed on small datasets remains a significant challenge.

The Grad-CAM visualisations (Fig. 2, Fig. 3) revealed that high-contribution scores were distributed throughout the brain rather than confined to specific localised regions. Clinically, *LIS1* and *DCX* mutations are known to exhibit distinct anterior–posterior gradients of severity regarding agyria and pachygyria (Pilz et al., 1998). However, lissencephaly is fundamentally a diffuse developmental disorder affecting global cortical lamination. The widespread Grad-CAM activation suggests that the 3D-CNN captures global morphological characteristics, including reduced sulcation, diffuse cortical thickening, and secondary alterations in whole-brain shape or ventricular volume, rather than relying solely on localised regional features.

Furthermore, resizing all MRI volumes to a uniform spatial resolution (64 × 128 × 128 voxels) may have biased the network toward extracting global geometric features over micro-level localised textures. Given that the model achieved a mean accuracy of 78.4% and exhibited widespread activation patterns in the Grad-CAM visualisations, these findings suggest that a holistic feature-extraction strategy is highly effective for causative gene identification in lissencephaly. Such an approach may enable evaluation of global patterns of cerebral development associated with specific genetic abnormalities and provides a complementary perspective to conventional assessments of localised disease severity. Although higher resolutions (e.g., 128 × 128 × 128) may capture finer structural details, they may also introduce interpolation-related artefacts during resampling. To maintain data integrity and ensure that the models focus on authentic biological features rather than processing-induced noise, we prioritised a resolution that balanced spatial detail and computational stability. Future research using high-field MRI or high-memory computing environments may enable evaluation of higher-resolution data while maintaining data integrity.

A limitation of the current methodology is the absence of standard neuroimaging preprocessing steps, such as N4 bias field correction, intensity normalisation (e.g., z-score matching), and skull-stripping. Although training directly on raw, resized volumes enabled evaluation under unaltered clinical conditions, the models may have partially learned scanner-specific artefacts or background noise. Future studies should incorporate standardised preprocessing pipelines to harmonise multicentre data and further improve model generalisability.

The external validation using the open-data sample provided additional insight into model robustness. Among the evaluated architectures, VGG16 demonstrated the highest success rate, correctly identifying the *DCX* mutation in both imaging modalities in five of six folds. This finding reinforces the observation that, under the current dataset-size constraints, relatively shallow architectures may provide more stable performance than deeper models.

However, the variable performance observed across architectures and imaging modalities (T1 vs. T2) highlights a critical challenge for clinical deployment. Such variability may reflect domain shifts arising from differences in signal-to-noise ratio (SNR), scanner protocols, and age-related brain developmental characteristics between the external 5-year-old patient and the training cohort. Addressing these generalisation gaps is essential for real-world implementation. As highlighted by recent trustworthy AI frameworks for healthcare (Li et al., 2026), robust human–AI collaboration and adaptive deployment strategies will be crucial for the clinical translation of automated diagnostic support tools.

## 5 Conclusions

In this study, we developed a causative gene identification model using 3D-CNNs trained on a brain MRI dataset to computationally determine the morphological characteristics associated with different genetic causes of lissencephaly. Our findings can be summarised as follows.

(1) A 3D-CNN model based on the ResNet18 architecture achieved a mean accuracy of 78.4% in a three-class classification task involving *LIS1*, *DCX*, and other genetic mutations.
(2) By implementing a prediction probability threshold of 0.9, the average precision across all models improved by 4.84% for the *LIS1* class and 4.67% for the *DCX* class compared with standard classification.
(3) Grad-CAM analysis demonstrated that the model's high-contribution regions were not confined to specific localised areas. Instead, features were extracted holistically from the brain MRI volumes.
(4) Applying the trained models to open-data samples showed that 15 of 78 model instances correctly identified the *DCX* mutation in both T1w and T2w MRI modalities.

The results of this study provide a foundation for improving accuracy through more advanced image-processing architectures. Furthermore, the findings suggest potential applications of transfer learning using larger MRI datasets from other neurological disorders, ultimately contributing to the development of automated diagnostic support tools for rare genetic diseases.

## Ethics statement

This study was approved by the Institutional Review Board (IRB) for Research Involving Human Subjects at Showa Medical University (Reference Number: G220-N, approved on October 1, 2025). Written informed consent was obtained from all participants.

## Data and code availability

The code and trained models used in the experiment are available online in Mendeley Data (doi: https://doi.org/10.17632/ybdxcbwp74.1). Please note that the raw training data are not publicly available because they contain sensitive personal information. However, researchers are encouraged to utilize the released pretrained models and referenced open-data samples.

## Declaration of generative AI and AI-assisted technologies in the manuscript preparation process

During the preparation of this work, the author(s) used Google Gemini in order to improve the language flow, ensure grammatical correctness, and format the manuscript responses. After using this tool, the authors reviewed and edited the content as needed and take full responsibility for the content of the publication.

## Funding

This study was supported by KAKENHI 24K11055 (MK and AY) and 21K12148 (AY); the Ministry of Health, Labor, and Welfare Research Program on Rare and Intractable Diseases under grant number JPMH23FC0201 (MK); and the Showa Medical University Intramural Joint Research Grant for Comprehensive Partnership Agreement (MK).

## CRediT authorship contribution statement

**Naoki Takahashi:** Investigation, Methodology, Software, Visualization, Writing – original draft. **Yoshihiro Sato:** Conceptualization, Data curation, Methodology, Supervision, Validation, Visualization, Writing – original draft. **Mitsuhiro Kato:** Data curation, Funding acquisition, Resources, Writing – review & editing. **Atsuko Yamaguchi:** Funding acquisition, Project administration, Writing – review & editing.

## Declaration of competing interest

The authors declare that they have no known competing financial interests or personal relationships that could have appeared to influence the work reported in this paper.

## Data Availability

The MRI data used for training is not publicly available, but we have released a trained model that ensures reproducibility and has been validated using open data.

## Abbreviations

2D, two-dimensional; 3D, three-dimensional; 3D-CNN, 3D convolutional neural network; AUPRC, Area Under the Precision–Recall Curve; CNN, convolutional neural network; FLAIR, fluid-attenuated inversion recovery; FOV, field of view; Grad-CAM, gradient-weighted class activation mapping; IRB, Institutional Review Board; MRI, magnetic resonance imaging; NMD, neuronal migration disorder; SBH, subcortical band heterotopia; SNR, signal-to-noise ratio; TE, echo time; T1w, T1-weighted; T2w, T2-weighted; TR, repetition time.
