# Supplementary material for: Deep learning-based identification of causative genes in lissencephaly using 3D-MRI volumetric datasets
Source: Neuroimage Rep. 2026 Jun 23;6(3):100375. doi: 10.1016/j.ynirp.2026.100375 (PMC13315807; doi:10.1016/j.ynirp.2026.100375)
Supplement: Multimedia component 1 [file mmc1.docx]

**Supplementary material**

**Supplementary Table S1:** Distribution of genetic subtypes included in the ‘Other’ lissencephaly category

| Genetic Subtype (Gene) | Number of Patients | Number of MRI Volumes |
| --- | --- | --- |
| ARX | 6 | 10 |
| WDR62 | 3 | 8 |
| DYNC1H1 | 3 | 8 |
| TUBB2A | 1 | 7 |
| FOXG1 | 1 | 6 |
| TUBB2B | 1 | 4 |
| KIF11 | 1 | 4 |
| CUL1 & WDR37 | 1 | 3 |
| ASPM | 1 | 2 |
| DARS2 | 1 | 2 |
| PNKP | 1 | 2 |
| RTTN | 1 | 2 |
| WDR81 | 1 | 2 |
| LAMC3 | 1 | 1 |
| Total (Other Category) | 23 | 61 |
